# The Royal College of Psychiatrists Physician Associate Inceptorship Programme: Developing Educational Programmes to Support the Integration of This New Role in Psychiatric Services

**DOI:** 10.1192/bjo.2022.136

**Published:** 2022-06-20

**Authors:** Pranav Mahajan, Helen Crimlisk, Chris Kenworthy

**Affiliations:** 1 Health Education Yorkshire & Humber, Sheffield, United Kingdom; 2 Royal College of Psychiatrists, London, United Kingdom

## Abstract

**Aims:**

Physician associates (PAs) are becoming more commonplace in psychiatric services in the UK to help address long term workforce difficulties. In 2019, the NHS Long Term Plan detailed a commitment to transforming mental health care in England recognising that services were not meeting current or future increase in demand. Health Education England's (HEE) report, Stepping Forward to 2020/21: The Mental Health Workforce Plan for England, described a longer-term strategy to expand the mental health workforce, including recruiting 5,000 people into ‘new roles’ including physician associates. The NHS Mental Health Implementation Plan 2019/20–2023/24 stated an aim of recruiting 140 PAs to the workforce over five years in addition to the requirements specified in the HEE report. HEE and the Royal College of Psychiatrists (RCPsych) have sought to support the integration of PAs into psychiatric teams through the development of the Inceptorship programme. The aim was to develop a bespoke training programme for PAs to bridge the gap between university and working in mental health to be rolled out nationally.

**Methods:**

Since 2018, Sheffield Health and Social Care Trust (SHSC) have been providing an Inceptorship Programme for PAs at the trust. Unlike with trainee doctors, there was no curriculum that could be followed. The programme covers the aetiology, diagnosis and management of common psychiatric problems, communication skills and reflective practice. This programme has provided the basis for the RCPsych Inceptorship Programme supported by HEE.

**Results:**

The SHSC programme has been well received by the 11PAs that have been through the programme, with all PAs recommending other mental health organisations take a similar approach. There have been many additional benefits of the sessions. They allow PAs to gain peer support and it has been a forum to raise issues which often arise when integrating new roles into pre-existing MDTs.

**Conclusion:**

The RCPsych Inceptorship Programme is a PA specific educational programme. It is an important tool in addressing the gap between variable mental health experience as a student (which is limited to a 3-week placement and is variable in content) and working in a psychiatric setting. RCPsych and HEE recommend that all mental health organisations employing PAs implement an inceptorship programme based on the work carried out at SHSC. These should comprise of regular, protected sessions that provide PAs with bespoke mental health training to support their integration into psychiatric multidisciplinary teams. HEE have agreed to provide funding to help organisations facilitate it.

